# Acromioclavicular joint dislocation: a novel surgical technique for acromioclavicular joint reduction with coracoclavicular ligament reconstruction and anatomic conoid ligament reconstruction

**DOI:** 10.1016/j.xrrt.2023.12.001

**Published:** 2024-01-12

**Authors:** Ronald Navarro, Michael Kody, Michael Chapek, Kristen Combs

**Affiliations:** a Department of Orthopedic Surgery, Southern California Permanente Medical Group, Harbor City, CA, USA; b Kaiser Permanente Bernard J. Tyson School of Medicine, Pasadena, CA, USA; c Department of Orthopaedic Surgery, Harbor-University of California, Los Angeles Medical Center, Torrance, CA, USA

**Keywords:** Acromioclavicular joint dislocation, Conoid ligament, Trapezoid ligament, Coracoclavicular ligament, Shoulder separation, Distal clavicle fracture, Suture button

The management of acromioclavicular (AC) joint dislocations remains controversial. Generally, Rockwood type 1 and 2 injuries are treated conservatively, and type 4, 5, and 6 injuries are treated surgically.^13^ There is no clear consensus on the management of type 3 injuries, and operative vs. nonoperative management is left to the surgeon’s discretion.^1^^,^^13^

The AC ligament and the coracoclavicular (CC) ligament complex work in conjunction to stabilize the AC joint of the shoulder. The AC ligament is a multidirectional AC joint stabilizer, and in the small, physiologic loads of everyday activities, it contributes nearly two-thirds of the AC joint stabilization to superior displacement.^7^ The CC ligaments become more important in resisting displacement of the AC joint at larger loads, such as those sustained during injury.^7^ At these higher loads, the conoid ligament has the greatest contribution to stability in most directions of loading.^7^ As the most common direction of clavicular displacement in an AC joint dislocation is superior and posterior, proper reduction of the AC joint and reconstruction of the CC ligament is paramount for AC joint stabilization.^7^

Most surgical techniques rely on CC ligament substitution to restore AC joint stability.^6^ Today, many suture button-based fixation methods use a single point of fixation in the distal clavicle, but newer implant designs seek to mimic the anatomic insertion of the CC ligaments by utilizing two separate clavicular tunnels for the implant.^8^^,^^9^ The use of two clavicular tunnels is thought to better replicate native CC ligament anatomy and improve stability when compared to single-point fixation.^8^^,^^11^

While dual clavicular limb fixation methods may better restore the distal clavicle to its native position, the described techniques do not account for the obliquity of the native CC ligaments^11^ Specifically, the conoid ligament attaches posteromedial on the clavicle and progresses anterolaterally to its insertion on the coracoid, and this obliquity likely contributes to the multidirectional stabilizing ability of the conoid.^5^ Previous attempts at recreating the conoid ligament stabilization vector through a more posterior clavicular tunnel placement have been associated with postoperative clavicle fractures.^16^ In this case series, it is hypothesized that the larger tunnels needed for graft fixation led to cortical breaching and construct failure when the conoid tunnel was placed too posteriorly.^16^ Furthermore, larger tunnels decrease the bone bridge size between the clavicular fixation points, and this decreased bone bridge size has been associated with clavicular fracture.^6^

We propose a novel approach to AC joint reduction and reconstruction using the Arthrex Twin Tail TightRope suture implant (Arthrex Inc., Naples, FL, USA) and an allograft loop construct in the setting of chronic AC joint dislocation. This technique seeks to better approximate the posterior-medial clavicular insertion of the conoid ligament in both the implant and allograft fixation. The medial tunnel of the suture implant is drilled with posterior to anterior obliquity to replicate the conoid ligament insertion and thus improve multidirectional stability. After the suture construct is placed and the AC joint is reduced, an allograft loop is then placed to approximate the anatomic relationship of the CC ligaments and to increase sagittal plane stability. This guide describes a novel approach to AC joint reduction and CC reconstruction and reviews the advantages and disadvantages, as well as the pearls and pitfalls of this novel technique (Table I; Table II).

**Table I:** Pearls and pitfalls of the described technique.

**Pearls**
Superior extent of the incision should be slightly more medial than traditionally described to facilitate clavicular exposure.
L-shaped hockey stick incision tracing medial to AC joint and then deltoid insertion just over clavicle allows for deltoid insertional repair.
Subtle anteriorization of the clavicle is critical to the reduction of many type 3 injuries.
Medial clavicular tunnel tightrope is placed 4.5 cm from the distal clavicle or at conoid insertion to best replicate native biomechanics.
Medial clavicular tunnel is angled in a posterior to anterior oblique path in approximately 45 degrees of posterior-to-anterior angulation from true vertical.
Surgeon must place allograft slightly anterior to the base of the coracoid to allow adequate space posteriorly for the tightrope construct.
Ensure coracoid tunnel at base is bicortical so coracoid button can be inserted and flipped on the undersurface of the coracoid.
**Pitfalls**
Failure to anteriorize the clavicle in relation to its displaced location may result in malreduction.
Do not drill allograft tunnel directly toward the suture button fixation, as drill can cut suture fixation.

*AC*, acromioclavicular.

**Table II:** Advantages and disadvantages of the described technique.

**Advantages**
Increased replication of native ligamentous insertion through implant fixation and allograft location.
Allograft supplementation with total of three drill tunnels.
No use of screw or interference fixation.
Anteriorization of clavicle using a suture button-type construct with medial drill hole angled 45 degrees posterior to anterior from the other superior to inferior tunnels.
**Disadvantages**
There is a potential increased fracture risk with oblique clavicle drill tunnel placement and three drill tunnels in the distal clavicle.

## Surgical technique

### Patient evaluation

A complete history and physical examination must be performed before surgery. Skin quality overlying the surgical site should be noted. A thorough upper extremity neurologic exam including the musculocutaneous distributions and sensation about the surgical region should be documented. Further, the patient should be aware of the potential for sensory changes about the surgical region.

### Positioning and incision

**Figure 1:**
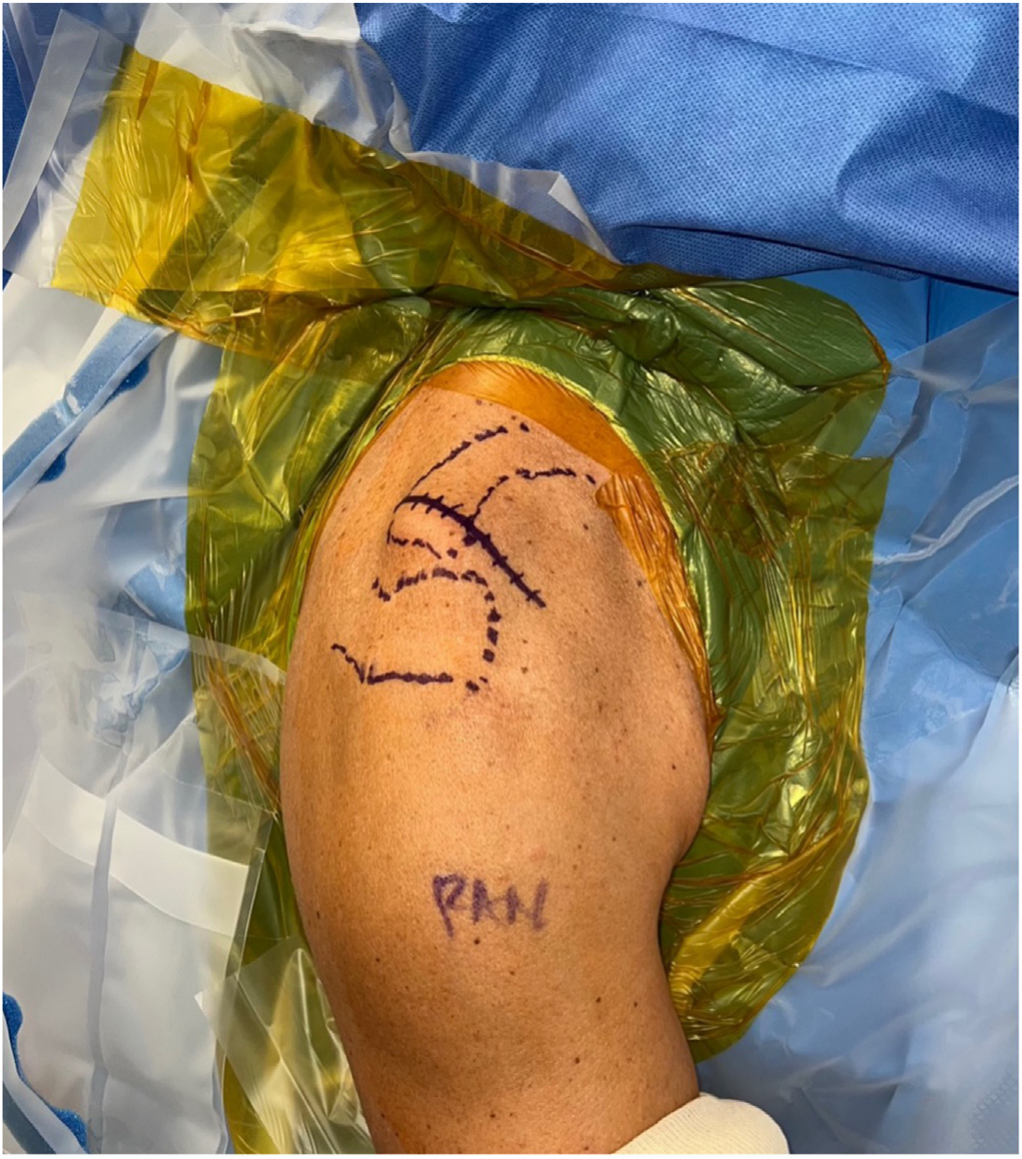
Saber-type skin incision, distal clavicle, and acromion marked. Incision begins at posterior clavicle centered over a point 1 cm medial to the AC joint. *AC*, acromioclavicular.

The patient is placed in the beach chair position with the operative extremity held in a motorized arm holder. The described saber approach to the scapula is slightly modified. The incision starts superiorly, approximately 1 centimeter (cm) medial to the posterior-lateral aspect of the distal clavicle, and terminates caudally at the coracoid (Fig. 1).

### Exposure

**Figure 2:**
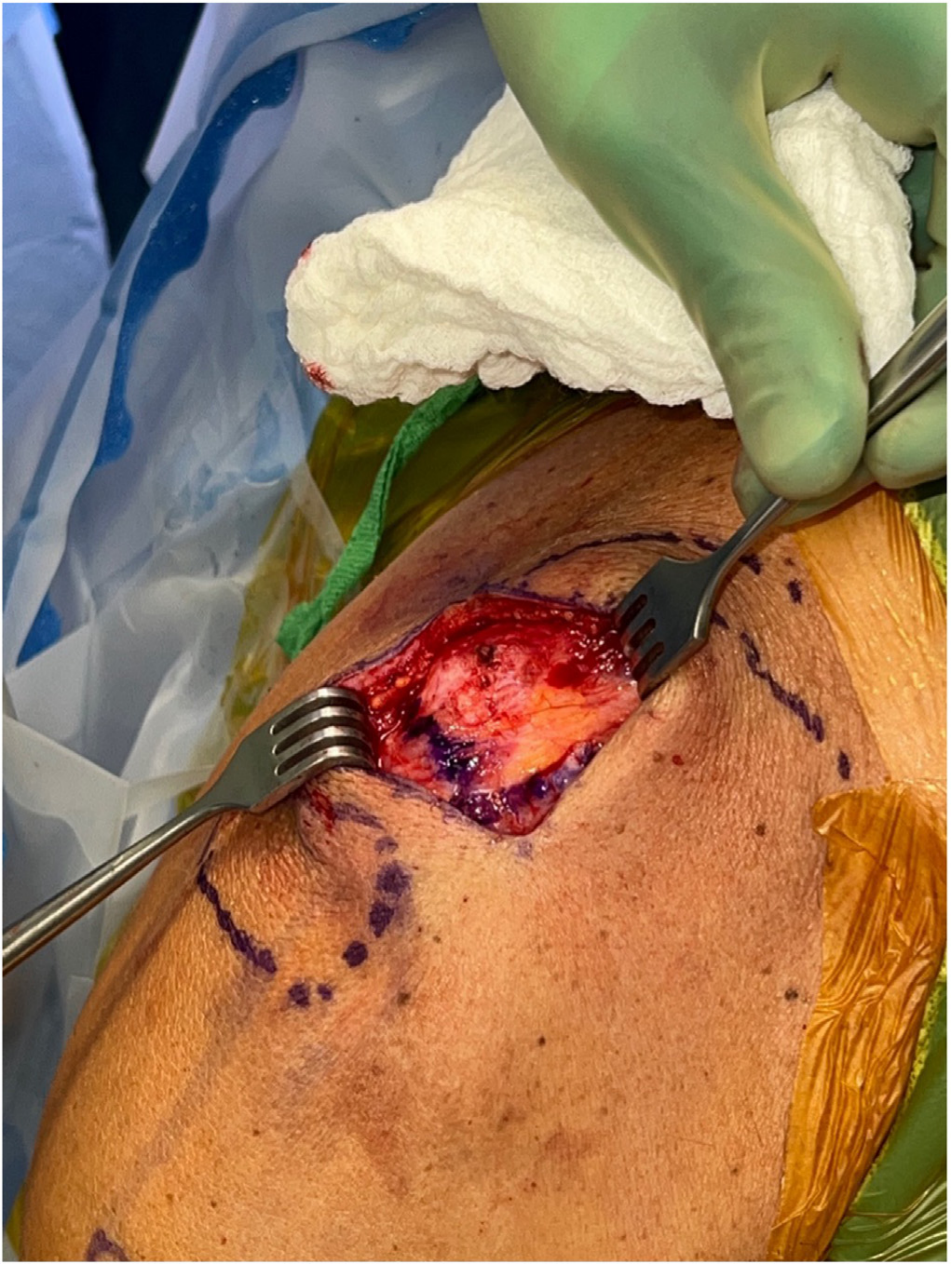
Hockey stick-type incision drawn from AC joint along anterior clavicle at deltoid insertion. *AC*, acromioclavicular.

**Figure 3:**
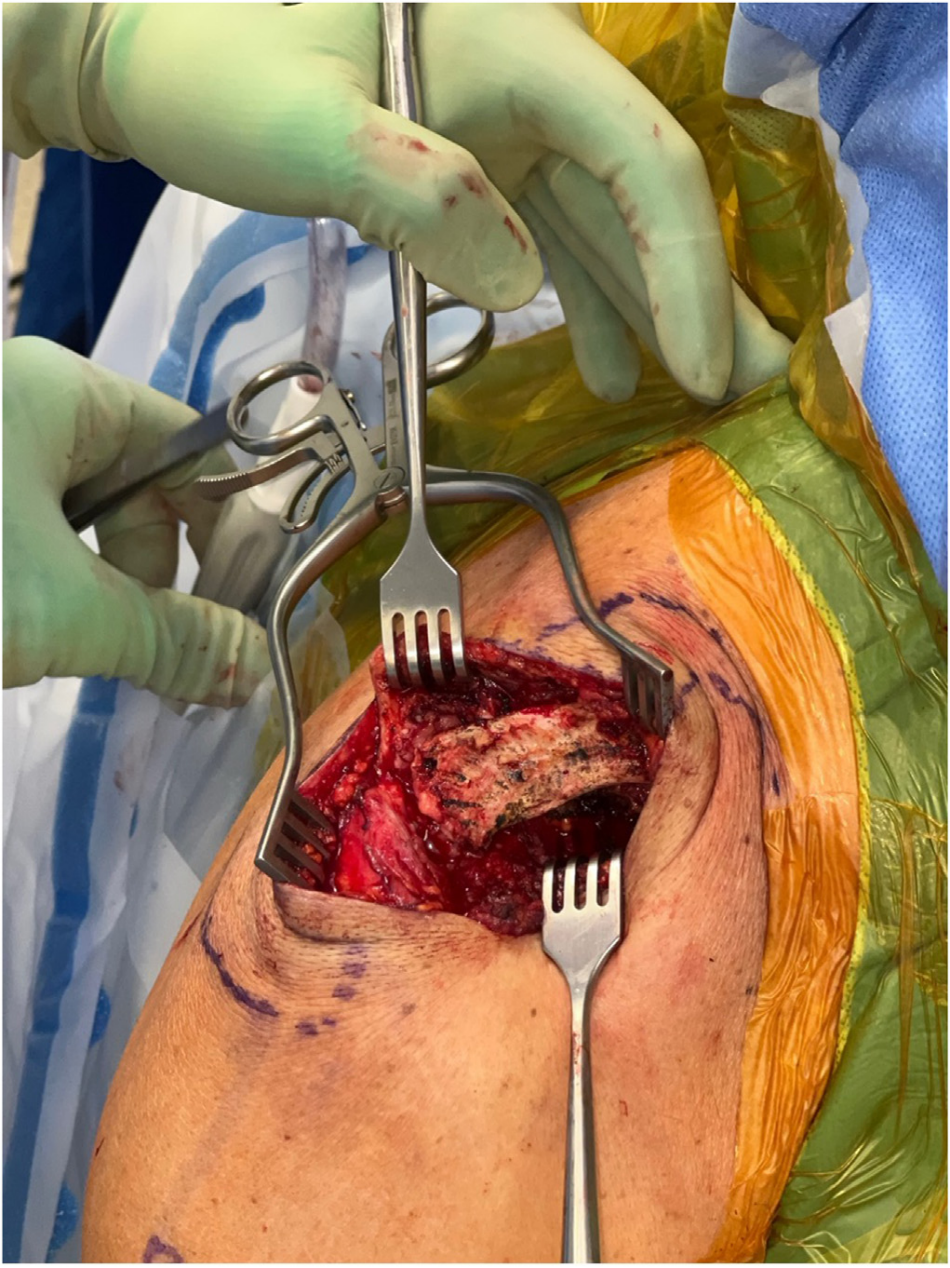
Distal clavicular and AC joint exposure with retraction of deltotrapezial fascial layer. *AC*, acromioclavicular.

**Figure 4:**
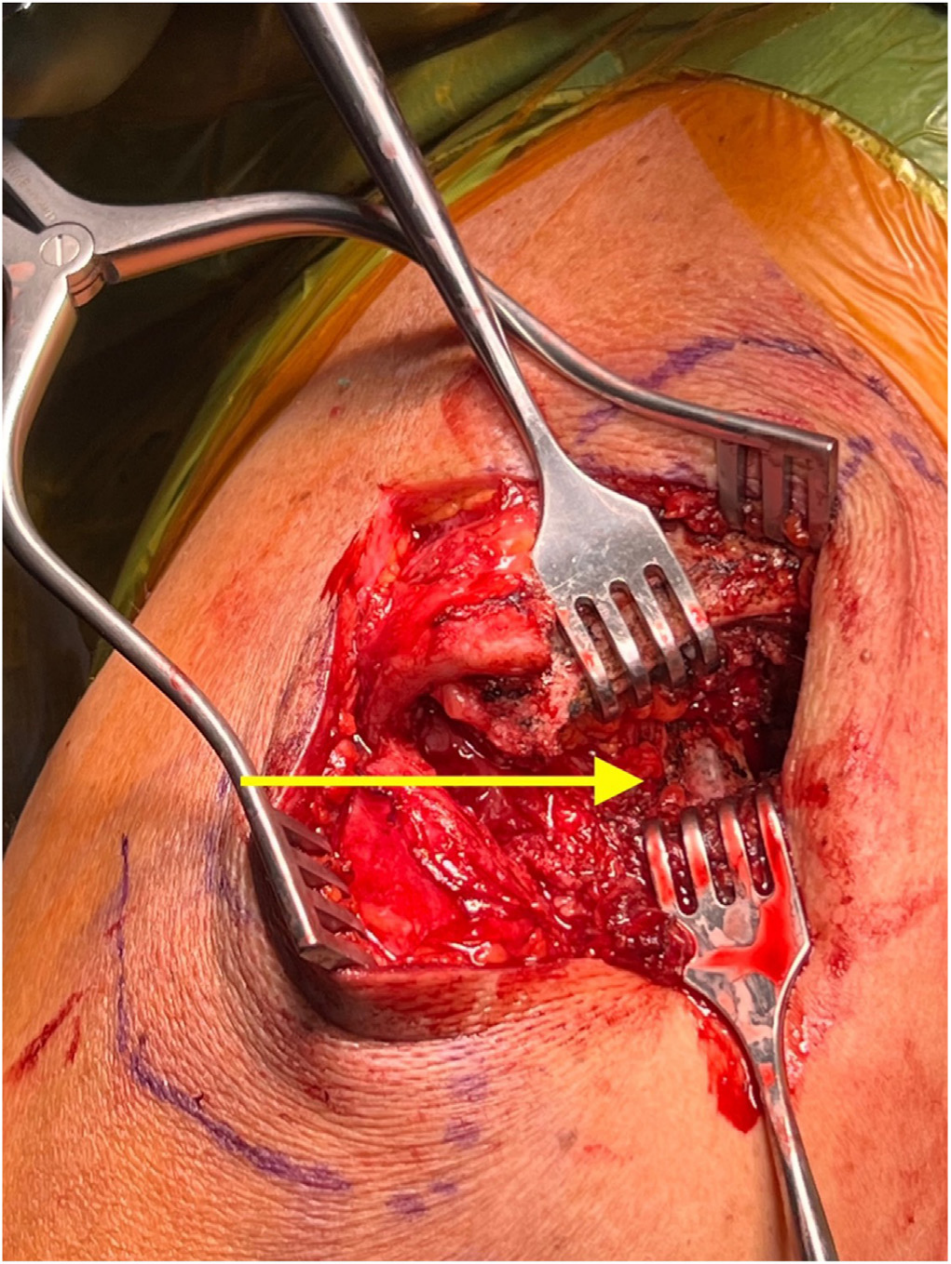
Exposure of coracoid process.

The deltotrapezial fascia is encountered after dissecting through the skin and subcutaneous tissue. After skin flap development, a hockey stick-shaped incision is utilized with the shorter limb extending along the AC joint and the longer limb following the anterior border of the clavicle (Fig. 2). Using electrocautery, the deltoid and trapezius are reflected off the clavicle with special care to leave repairable fascia on either side of the flap. Electrocautery is then used to expose the AC joint and 2-3 cm of the distal clavicle (Fig. 3). This dissection is continued inferiorly with the clavicle retracted posteriorly for adequate exposure of the coracoid base at the scapular body (Fig. 4). Often, there are CC ligament remnants that must be débrided off of the superior and more posterior aspects of the coracoid, where it emanates away from the scapular body. This is best achieved with electrocautery.

### Allograft preparation and augmentation

**Figure 5:**
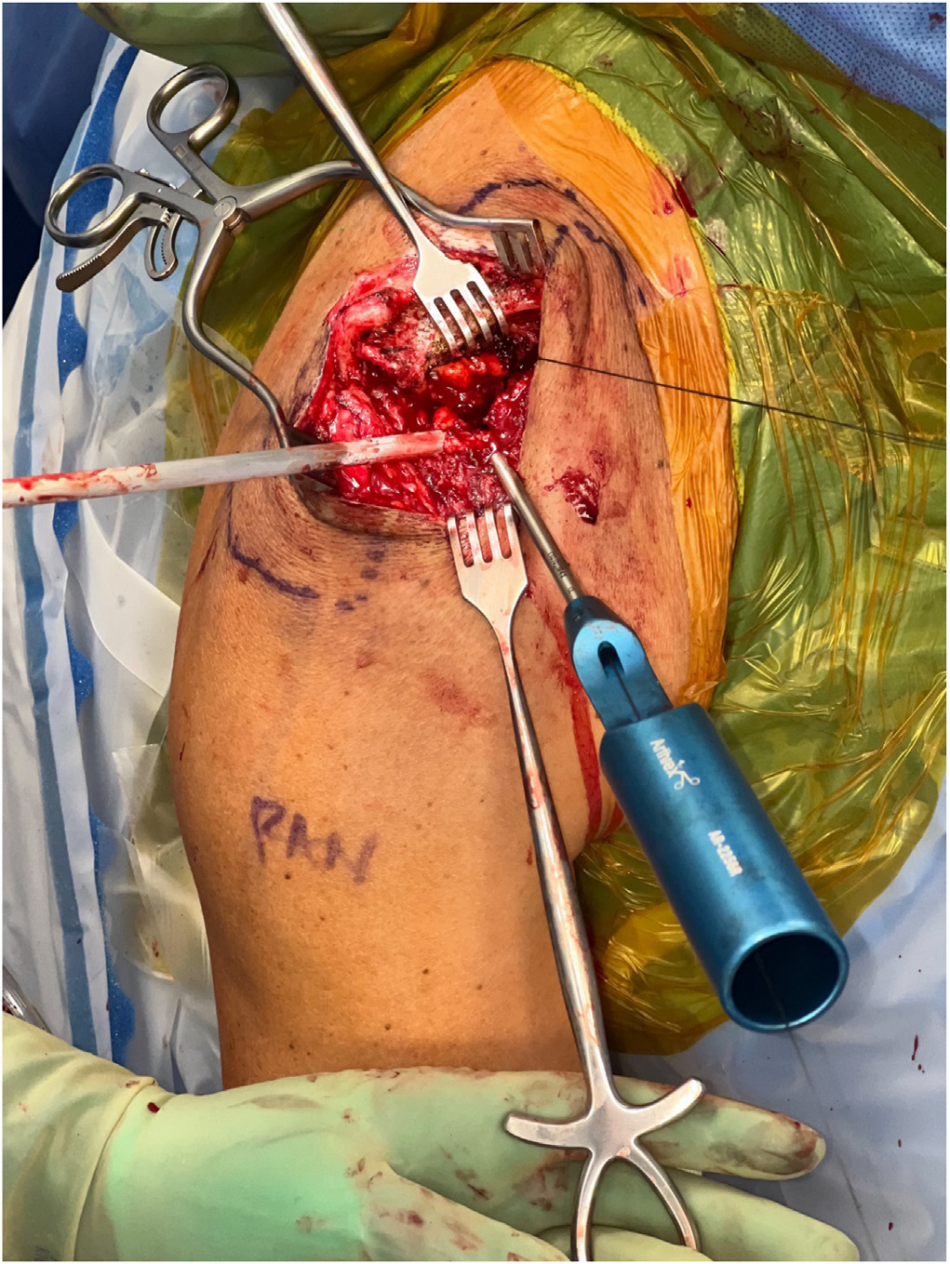
Suture passed under base of coracoid slightly anterior to planned position of suture button location. Medial limb of suture passer is seen as a black line across the chest.

Semitendinosus allograft is used for CC ligament reconstruction. Two sutures are placed through each end of the graft, and the graft is sized. We commonly use a 5-6-millimeter diameter allograft. The graft is then advanced under the coracoid using a looped suture lasso, right-angle clamp, or a commercially available passing device. Typically, small soft tissue windows must be created on the medial and lateral borders of the coracoid’s undersurface to gain entry for the passing device and then the graft. Final graft location should be slightly anterior at the coracoid base to allow adequate space for the suture button construct (Fig. 5). Once in an appropriate location, the graft ends are snapped and brought anteriorly away from the clavicle.

### Coracoid tunnel

**Figure 6:**
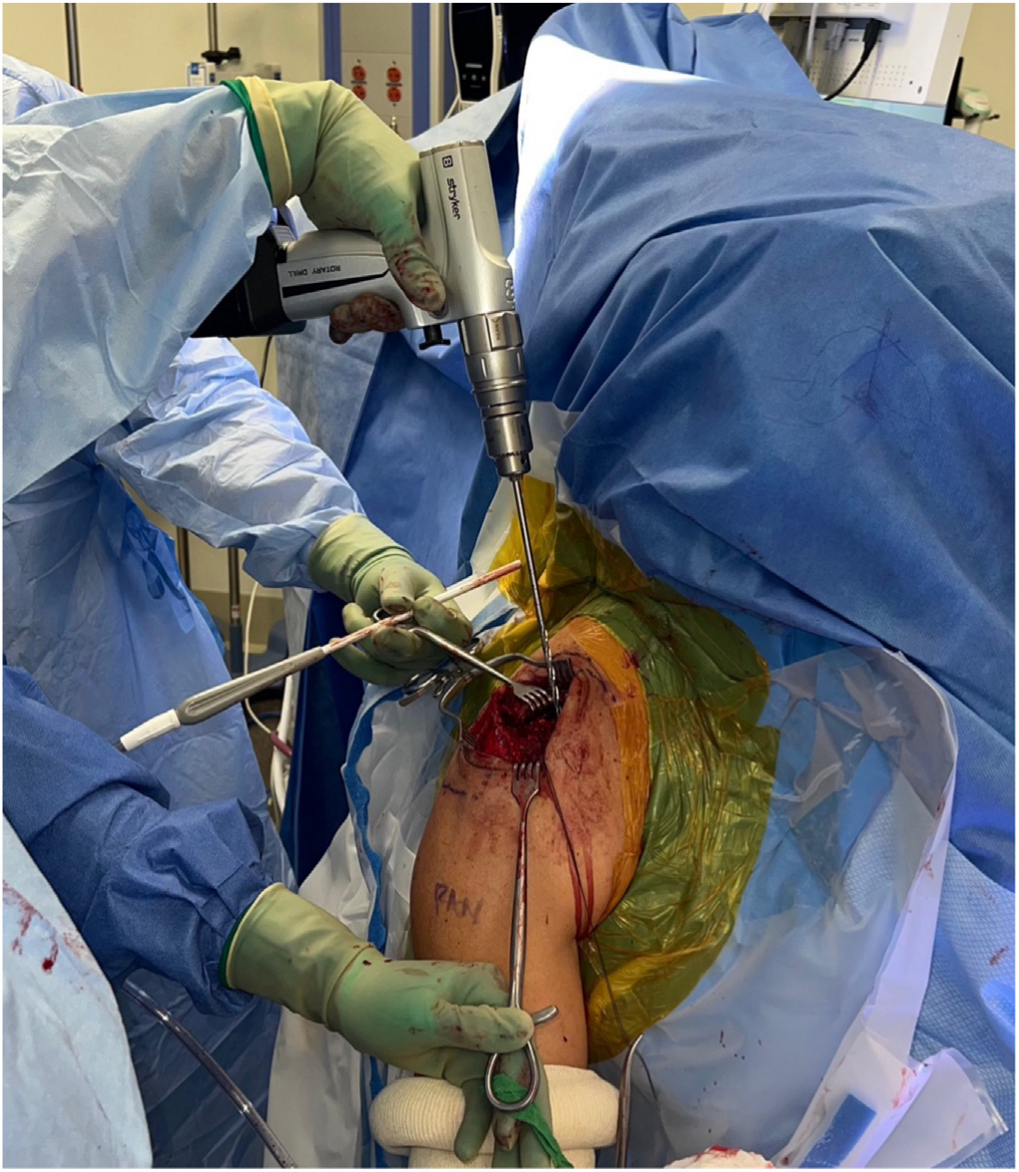
Trajectory of drill path for coracoid tunnel in relatively vertical plane.

**Figure 7:**
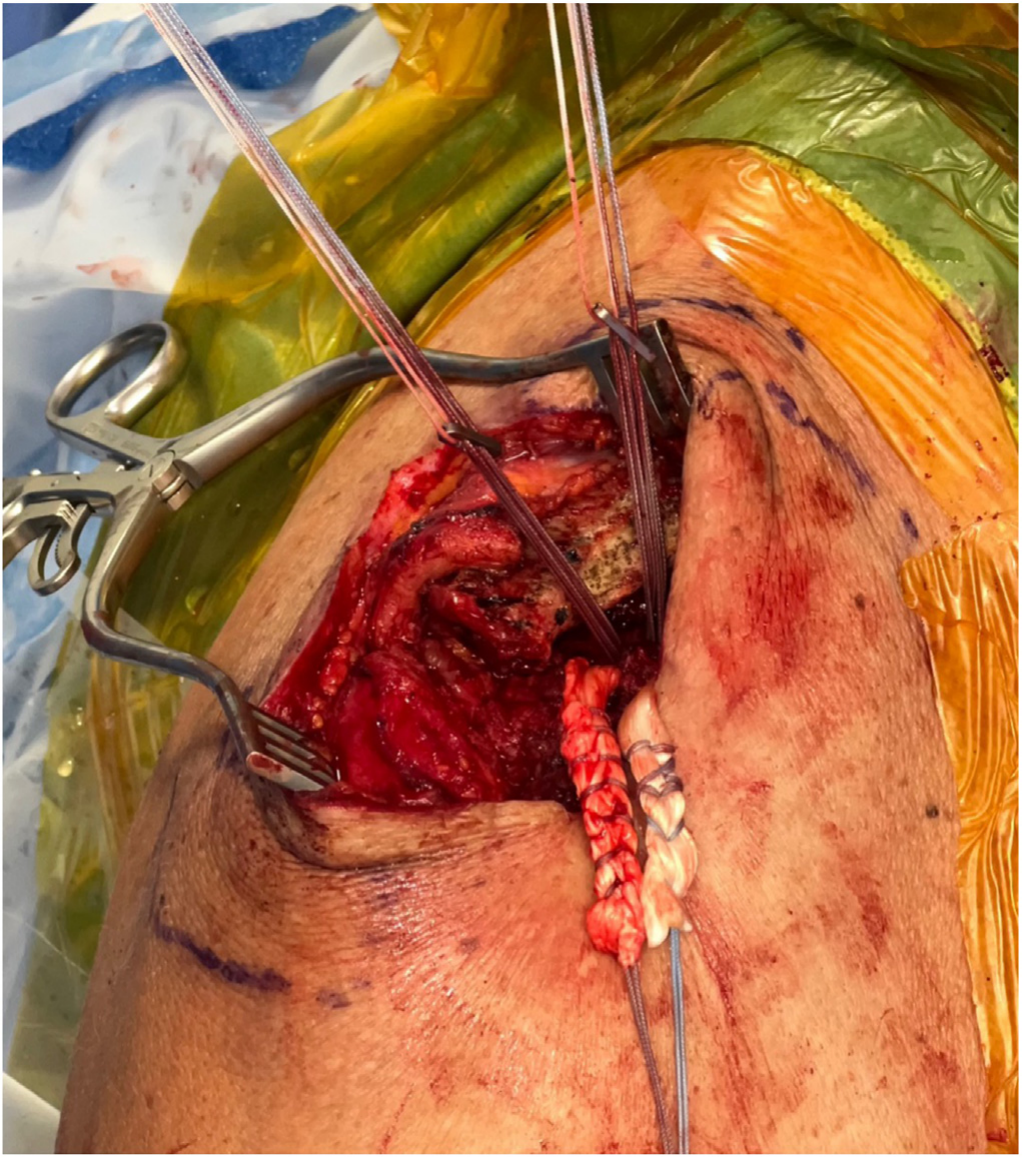
Allograft looped beneath base of the coracoid process anterior to now placed suture button at true base of coracoid process. The graft is brought anterior to allow implant fixation to the clavicle.

We utilize the Arthrex Twin Tail TightRope for our AC joint reduction and fixation. The coracoid tunnel is drilled with the guide pin placed at the base of the upper surface of the coracoid (ideally at its coronal apex) to ensure sufficient bone medially and laterally (Fig. 6; Video 1). Care must be taken to ensure bicortical fixation ensuring that the tunnel is not too far posterior and in the body of the scapula instead of passing through the undersurface of the coracoid. The tunnel should be just beyond the base of the coracoid, as fatigue fractures can occur if the tunnel is too anterior. Fluoroscopic guidance can help in these determinations. Care must also be taken to avoid the allograft, which is anterior, when placing the guide pin and drilling. Alternatively, this step can come before the “allograft passage under coracoid” step. The implant is then secured in the coracoid by placing the basilar suture button of the Arthrex Twin Tail TightRope through the superior coracoid tunnel and through the coracoid base. It is then flipped with the button guide (Fig. 7). A test to ensure the button has flipped to be horizontal is performed by pulling the sutures cranially. If the button is appropriately flipped, pulling on both of the clavicular suture limbs will result in the scapula essentially lifting away from the chest, and the button will not slip out of the coracoid tunnel.

### Clavicular tunnel creation/anteriorization

**Figure 8:**
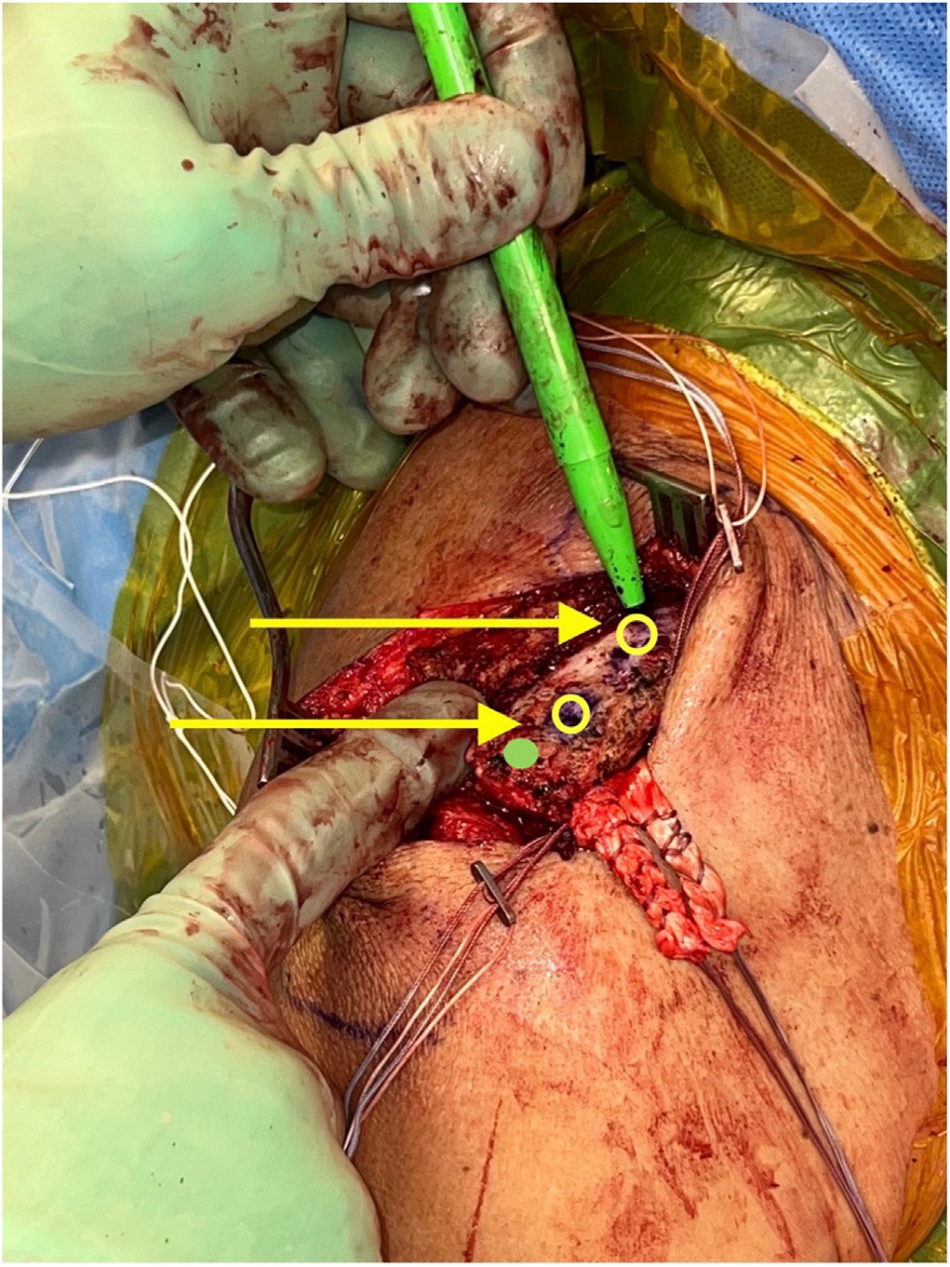
Location of medial and lateral clavicular drill tunnels for suture button fixation are indicated with yellow open circles. Note the posterior start point of the medial tunnel. Lateral allograft tunnel is indicated with green closed circle.

**Figure 9:**
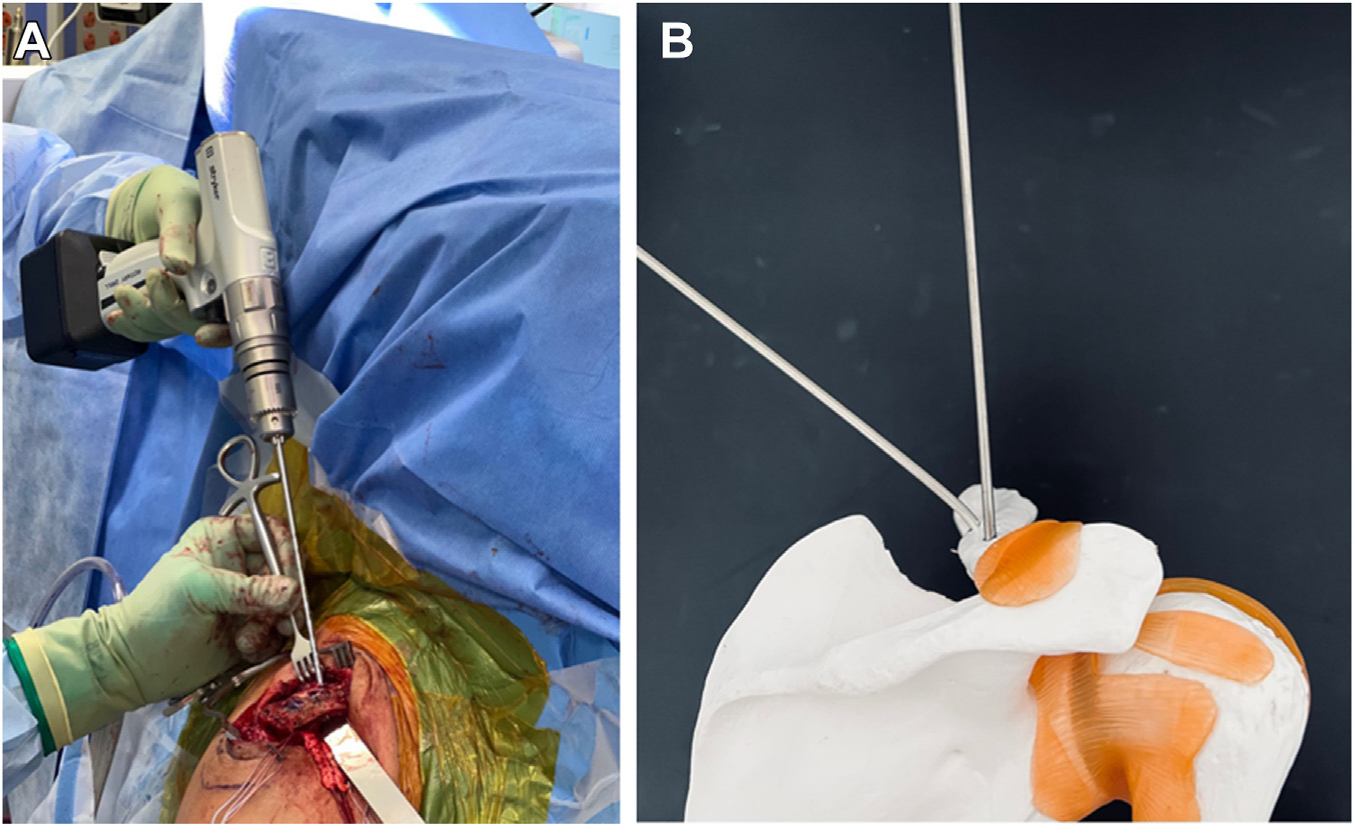
**(A)** Medial clavicular guide pin with 45-degree posterior to anterior angulation. **(B)** Model representing obliquity of medial drill path.

**Figure 10:**
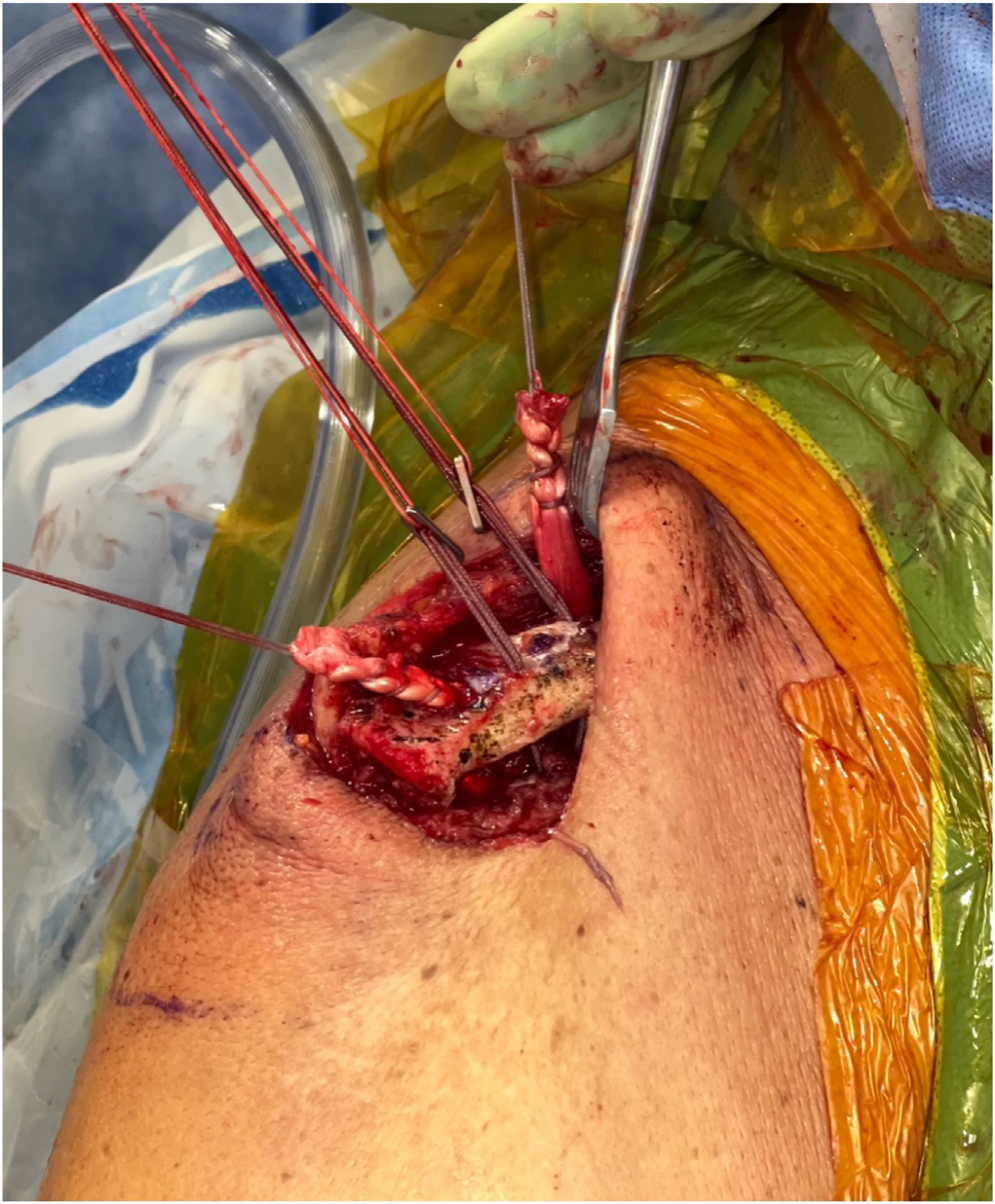
Implant passed through medial and lateral drill tunnel. Lateral arm of allograft passed through the most lateral tunnel. Medial arm of allograft is brought posterior to clavicle.

**Figure 11:**
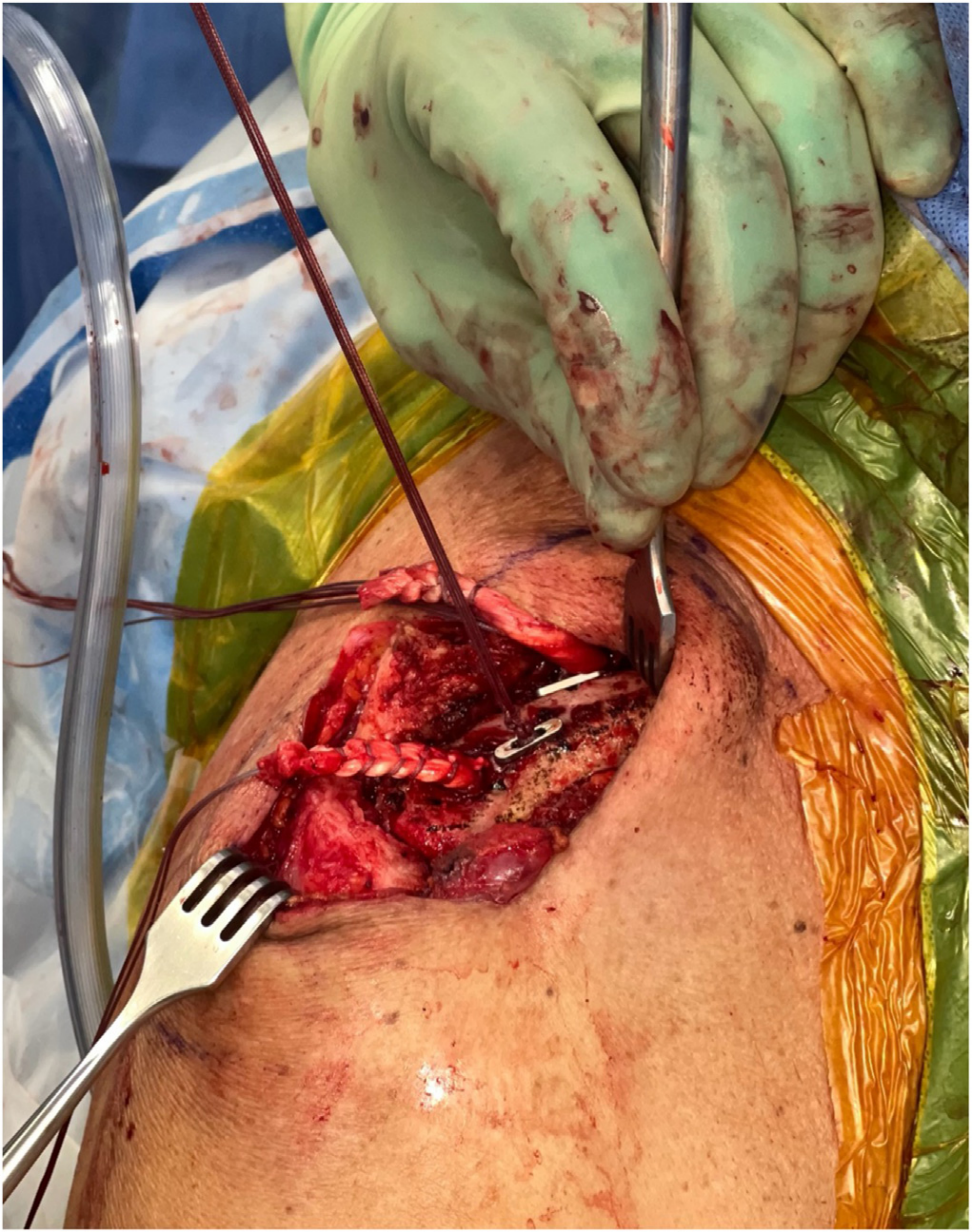
Suture buttons tightening with reduction of AC joint. Note the posterior medial suture button. *AC*, acromioclavicular.

**Figure 12:**
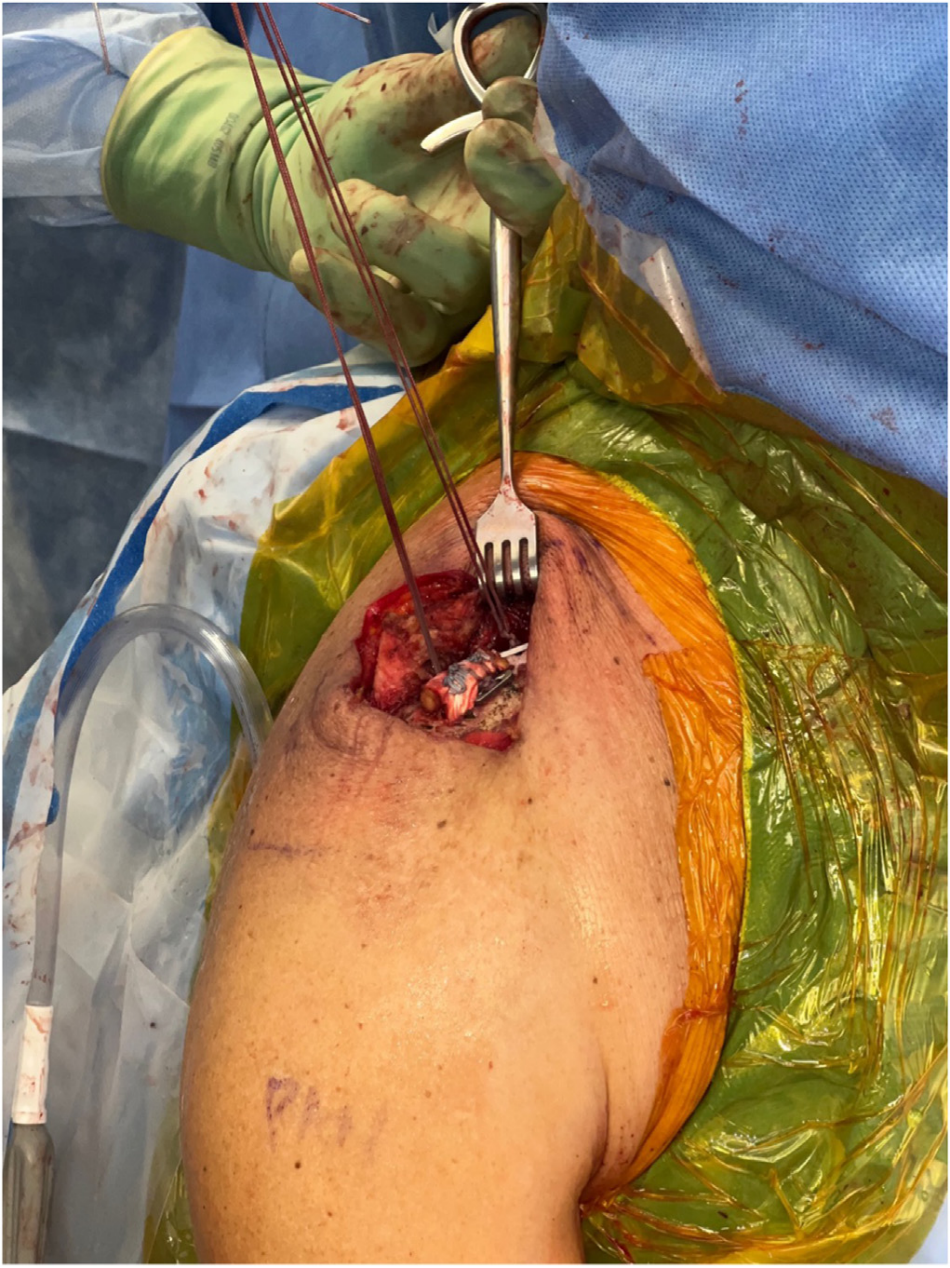
Final allograft fixation with each end tied to itself passing over the distal clavicle and suture buttons.

**Figure 13:**
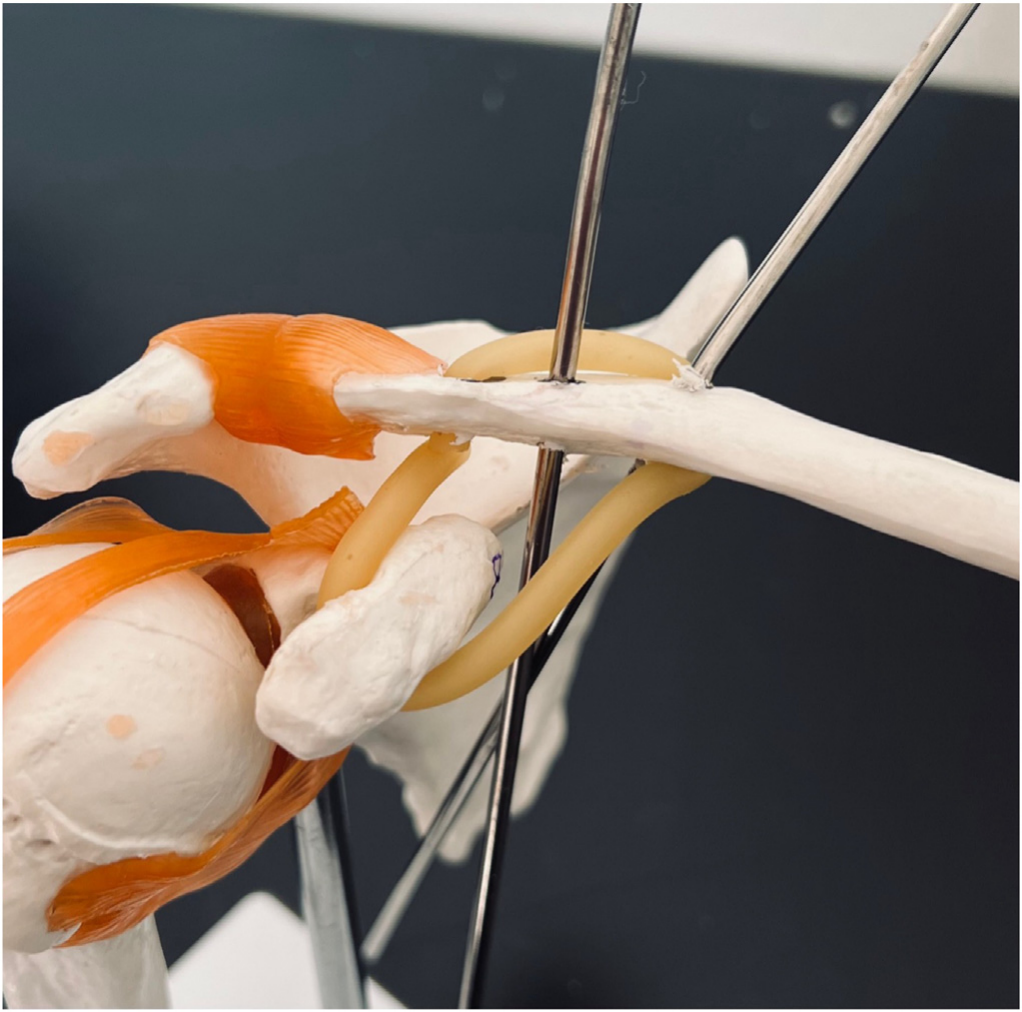
Mock-up of final construct and guide pin obliquity.

**Figure 14:**
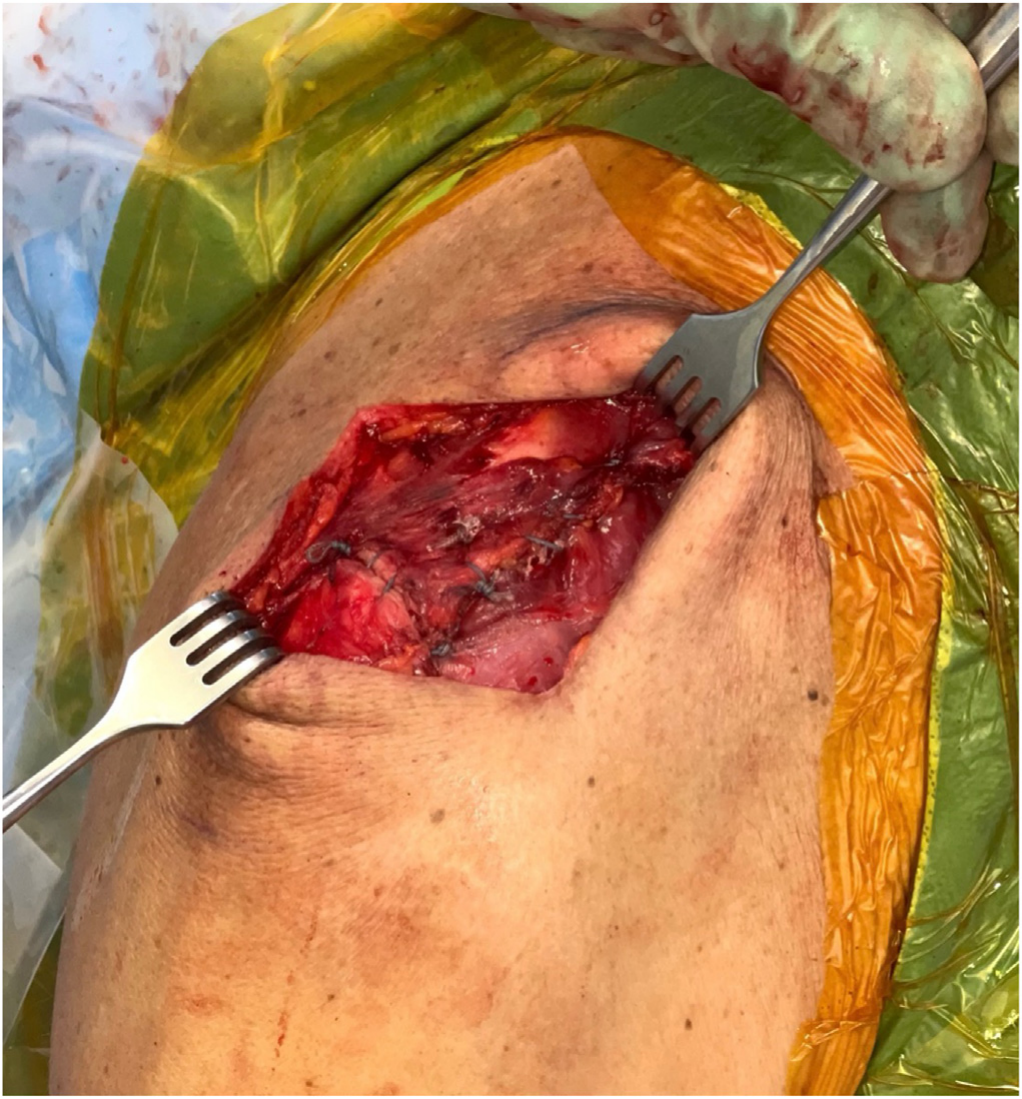
Hockey stick-type incision closure with repair of deltoid and deltotrapezial fascia.

The clavicular tunnels for the implant and graft are then marked (Fig. 8). An appropriately sized drill tunnel matching the diameter of the allograft is then placed on the lateral clavicle. This tunnel takes into account the two more medial clavicular tunnels to be made for the limbs of the suture construct. The allograft tunnel is approximately 2 cm medial from the AC joint and angled vertically. The implant clavicular tunnels are identified using native ligamentous anatomy or by approximating 3 cm and 4.5 cm from the most lateral portion of the clavicle. The lateral tunnel is drilled in the typical vertical fashion, centrally on the apical clavicle toward the coracoid base. The medial tunnel is placed slightly posteriorly on the clavicle and angled in a superoposterior to inferoanterior oblique path, approximately 45 degrees from true vertical (Fig. 9 *A* and *B*). It is important to clear soft tissue from under the clavicle at the tunnel orifices, as this may impede the passage of the implants and graft. The suture buttons are then advanced in a retrograde fashion (inferior to superior of the clavicle) through each tunnel. At this point, the graft is passed retrogradely through the most lateral tunnel. The medial end of the allograft is then advanced posterior to the clavicle—to replicate the conoid insertion—after creating a small pocket for passage (Fig. 10). The medial limb of the allograft should wrap posteriorly at the clavicle approximating the conoid insertion. The AC joint is reduced manually, then the medial button, followed by the lateral button, is sequentially tightened, and both sutures are tied with half-hitches to lock the implants on the clavicle (Fig. 11). This sequential tightening ensures the posterior to anterior translation (though the medial suture limb) is corrected before locking the final superior to inferior reduction. One- or two-millimeter over-reduction is acceptable to ensure that if any loss occurs, the AC joint remains largely reduced. The two graft ends are then sutured to one another using absorbable suture as they are laid over the superior implant buttons to aid in hardware coverage (Fig. 12). A replica of the allograft construct with pins demonstrating tunnel obliquity is shown (Fig. 13). The wound is then thoroughly irrigated. The sutures from the button implants are passed through the deltotrapezial fascia and loosely tied down to bring the fascia forward. The deeper hockey stick incision is closed over the clavicle using nonabsorbable suture to reapproximate the anterior deltoid (Fig. 14). This is followed by a layered skin closure.

### Postoperative protocol

**Figure 15:**
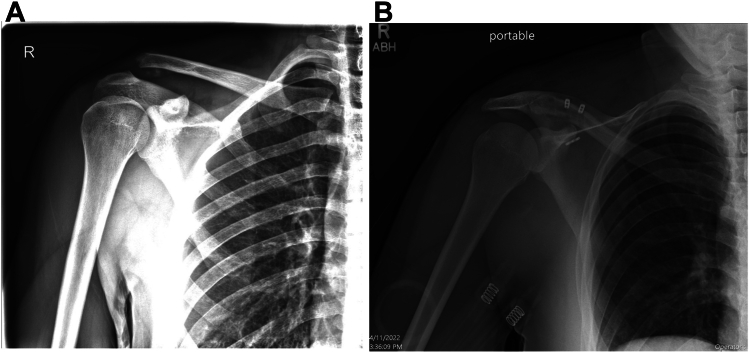
**(A)** Radiographs demonstrating a right-type III AC joint separation. **(B)** Radiographs demonstrating maintenance of AC joint reduction. *AC*, acromioclavicular.

The patient is placed in a shoulder immobilizer and is nonweight-bearing for six weeks. Physical therapy is initiated at two weeks with passive range of motion, progressive active assisted range of motion, and then finally, active range of motion in all planes. In the first few weeks, the only position of avoidance is extreme adduction with the arm in 90 degrees of forward flexion to avoid AC joint compression. Strengthening occurs in a progressive fashion with avoidance of anterior deltoid resistive strengthening beyond the weight of the arm for 4-6 weeks while the deltotrapezial fascia heals. Thereafter, anterior deltoid resistive strengthening can proceed. Functional and sport-specific motions and maneuvers are initiated at 2-3 months postoperatively. Preoperative and postoperative radiographs demonstrate maintenance of AC joint reduction (Fig. 15 *A* and *B*).

## Discussion

The management of AC joint dislocations remains a difficult surgical problem with over 60 operative techniques described in the literature.^16^ Further complicating surgical treatment options is the relatively high complication profile, including loss of reduction, need for hardware removal, and coracoid or clavicular fracture.^10^ As loss of reduction is the most frequent postoperative complication, we believe that stable anatomic fixation is fundamental to improve patient outcomes.^10^

Our hybrid fixation method, with anatomic tunnel placement, utilizes the Twin Tail TightRope construct for AC joint reduction and the semitendinosus allograft for CC ligament reconstruction. The allograft provides additional anterior to posterior stability to the reconstruction. Alternative options for AC joint dislocation have numerous drawbacks. Hook plate fixation is an exceedingly stable construct, but this stability is at the cost of a nonanatomic reduction and routine secondary surgery for hardware removal.^14^^,^^3^ Further, a recent randomized clinical trial found no clinical superiority to hook plate fixation vs. nonoperative management for complete AC joint dislocations.^4^ Recently, suture-based constructs have become more common for management of AC joint separation. Single-limb suture techniques do not inherently possess a stabilization vector to control anterior and posterior translation. Therefore, it has been hypothesized that double-limb fixation would better address AC joint stabilization.^2^ A direct comparison between the two methods in cadaveric models showed greater multidirectional stability using a dual-limb technique, yet their findings did not reach statistical significance.^2^ Interestingly, previous cadaveric biomechanical studies have demonstrated that dual-limb suture fixation is equivalent or stronger when compared to native ligaments.^17^ Additionally, a recent retrospective study comparing clinical outcomes between single-limb suture fixation and dual-limb fixation demonstrated no changes in patient reported outcomes but found significant radiographic loss of reduction in the single-limb group.^8^ They reported a complication rate of approximately 10% in the single-limb group compared to zero complications in the dual-limb group.^8^ In our technique, we utilize the stability of dual-limb suture fixation to maintain AC joint stability and reduction while the allograft definitively incorporates into the coracoid and clavicle. Further discussion of our technique may be found in the attached video (Video 2).

The use of allograft fixation is an important component of our technique. The senior author frequently utilizes this hybrid fixation construct in injuries older than 3 months or in patients over the age of 50. Still, these patients are evaluated on a case-by-case basis, and occasionally, these constraints are relaxed. In younger patients with more acute injuries, the addition of allograft may or may not be required. A recent retrospective review of surgically treated acute Rockwood type III-V injuries showed similar functional outcomes, complications, and revision rates when comparing AC joint reconstruction with and without allograft.^12^ While this study did not identify significantly different results between the fixation strategies, there was a statistical difference in patient age, time to operation, and injury severity, whereas the suture-fixation-only patients were younger, presented more acutely, and were more likely to have Rockwood type III injuries than the suture and allograft fixation patients. We suggest that in managing younger patients with acute injuries, the decision to proceed with allograft should be made intraoperatively after thorough evaluation of the integrity of the CC ligament tissue. Additionally, care should be taken during the approach and tunnel placement to preserve remnant CC tissue in case allograft supplementation is not necessary.

It is important to acknowledge that all tunnel-based allograft reconstruction methods are an imperfect way to recreate the broad insertions of the CC ligaments. This is in part because larger-diameter grafts better approximate native ligament size, but the larger-diameter tunnels required with this fixation weaken the clavicle and increase fracture risk.^15^ Therefore, in previously described methods, there is a tradeoff between the risk of fracture and anatomic graft placement. In our technique, the suture button fixation utilizes smaller tunnels, allowing the surgeon to place these clavicular tunnels in a more accurate anatomic location with decreased risk of fracture. Further, the suture button fixation reduces and stabilizes the AC joint as well as the CC three-dimensional spatial relationship. Once this is achieved, our allograft is placed in a way that approximates the broad conoid insertion without requiring a large drill tunnel in this vulnerable location. The allograft loop construct further adds sagittal plane stability to the AC joint reconstruction due to its posteromedial limb and anterolateral limb looping over the clavicle.

## Conclusion

This surgical technique for the management of AC joint injuries recreates the anatomic relationship of the CC ligaments on the clavicle using a suture-based implant and allograft. We believe that this technique results in a more stable construct while maintaining surgical efficiency and minimizing postoperative fracture rate. This technique can also be applied in the treatment of distal clavicle fractures, both with and without ligamentous reconstruction, depending on the fracture morphology. Finally, this method is reproducible and provides a reliable option for the surgical management of AC joint injuries.

## Disclaimers:

Funding: No funding was disclosed by the authors.

Conflicts of interest: The authors, their immediate families, and any research foundation with which they are affiliated have not received any financial payments or other benefits from any commercial entity related to the subject of this article.

## Supplementary Data

Video 1 AC Joint Reconstruction Technique Discussion of critical technical details and pearls with Dr. Ronald Navarro.

Video 2 AC Joint Reconstruction Technique Q and A Question and Answer format addressing the reasoning and implementation of this technique for AC joint reconstruction.
